# Enhancing the Quality and Utility of Content Analyses for Addictive Disorders

**DOI:** 10.3390/ijerph15071389

**Published:** 2018-07-02

**Authors:** Melvyn Zhang, Tracey Wing, Daniel S. S. Fung, Helen Smith

**Affiliations:** 1 National Addictions Management Service, Institute of Mental Health, Singapore 539747, Singapore; 2 Family Medicine and Primary Care, Lee Kong Chian School of Medicine, Nanyang Technological University Singapore, Singapore 308232, Singapore; h.e.smith@ntu.edu.sg; 3 Institute of Mental Health, Singapore 539747, Singapore; tracey.wing@mohh.com.sg (T.W.); daniel_fung@imh.com.sg (D.S.S.F.)

**Keywords:** M-health, content analysis, reviews, health applications, alcohol apps, Cannabis apps

## Abstract

The advances in Mobile Health (M-health) technologies has led to an increase in the numbers of alcohol and drugs applications on the commercial stores. Content analyses and reviews of applications to date have demonstrated that most of these applications are for entertainment or information purposes. More recent content analyses have identified common behavioural change techniques in substance applications. Nevertheless, there remain several limitations of existing content analyses and reviews of applications. There is an increasing prevalence of other substance-related disorders, such as that of stimulants and opioids, but the existing content analyses are limited to an analysis of alcohol and cannabis applications. Only two of the content analyses performed to date have attempted to identify applications that have their basis on a theoretical approach, based on the identification of behavioural change techniques or motivational techniques. There is a need to identify applications on the commercial stores that replicate conventional psychological interventions, or at least provide elements of conventional psychological interventions using behavioural change techniques that are integrated into the application. Further evaluative research could be done on these applications to determine if they are efficacious before using them for patient care. To address the limitation that existing content analyses have only focused on reviews of alcohol and cannabis applications, we propose for there to be updated content analyses for alcohol and cannabis, and new content analyses for other substances of abuse (such as opioids and stimulants). We like to suggest that future reviews consider keywords such as abstinence or recovery, and ones that relate to psychological therapies, such as self-determination or attention bias retraining, as commercial applications that have an underlying psychological basis might be categorised differently, under different keyword terms. We have evidence of how a better search strategy identifies previously unrecognised applications for attentional bias modification.

## 1. Introduction

M-Health (Mobile Health) refers to the use of mobile technologies, such as mobile phones and their accompanying applications for healthcare interventions [1]. The advances in M-health have led to there being a corresponding increase in the number of alcohol and drugs-related applications being marketed on the commercial stores. Smartphone applications are increasingly popular, as for the healthcare professionals, they serve as good tools for primary and secondary prevention of addictive disorders. Moreover, applications could serve as tools that could augment traditional interventions [2]. There has been an increase in the utilization of applications by individuals, as mobile applications help to overcome geographic barriers, and stigma associated with help seeking [2]. There are also economic advantages for individuals, given that the cost associated with using an application is usually minimal, with a significant proportion of the applications being free for individuals to download [2]. Donker T et al. (2013) [3] has, in their prior systematic review, highlighted that there are currently applications in the published literature that seek to intervene for psychiatric disorders, that of depression, anxiety, and substance abuse. However, only a limited number of evidence-based applications are made available on the application stores, and there remains a need for further evaluation of the scientific evidence for most applications. To our knowledge, there has been a series of content analyses undertaken to characterize substance-related applications on the stores, and to determine their underlying evidence base. Our aim is to synthesize the evidence arising from existing content analyses and to identify their limitations. By doing so, we can propose methods that could help bridge the gap in the current research literature.

## 2. Evidence from Existing Content Analyses

The commercial smartphone applications have been subjected to a series of content analyses, with the aims of providing an overview of what types of applications are available and identifying any underlying issues and inaccuracies in existing applications. The first content analysis of alcohol-related smartphone applications was undertaken by Weaver ER et al. (2013) [4], who evaluated 384 applications from Apple iTunes and Google Play store. The applications were identified using the search term “alcohol”, and only the top 250 applications on each store were included. A total of 50% of the sampled applications were entertainment applications, and only a minority (11%) were applications that provided information about health or methods to reduce drinking. Their assessment of the applications for blood alcohol concentration, which used data acquired from the Patron Offending and Intoxication in Night-time Entertainment Districts (POINTED) study, highlighted inaccurate estimates of blood alcohol concentrations levels when compared to the actual data acquired from breathalyser test [4].

Two years later, a review by Crane D et al. (2015) [5] of alcohol-related applications available in the United Kingdom sought to identify applications which used behavioural change techniques. As above, the applications were identified from both Apple iTunes and Google Play store using the keyword, “alcohol”, but only the initial 200 results were included in the study. A total of four separate searches between April to May 2014 were conducted, with 800 search results considered. A total of 662 unique applications were identified and, as Weaver ER. (2013) [4] had found, the minority (13.7%) of these applications were helping in alcohol reduction, with the majority (53.9%) being entertainment applications [5]. Crane et al. (2015) [5] found that the most commonly utilised behavioural change techniques identified were enabling individuals to self-record their behaviours (54.1%), followed by the provision of information of excessive usage of alcohol (42.6%), feedback on performance (41.0%), and options for alternative and subsequent support (24.6%) [5]. In 2017, but relating to applications identified in 2015, Hoeppner BB et al. (2017) published a third content analysis [2]. Using the keywords, “drinking”, “drink”, “alcohol”, “alcoholism”, and “sobriety”, 266 applications were identified in the Android store. Their content analysis sought to determine if alcohol applications provided any form of tailored messages as well as well as describing their standard functionalities [2]. They found almost two-thirds of applications (60%) provided some mechanism for tailoring of feedback. The most common form of tailored messages was based on drinking, demographics, and time [2], but only a minority provided tailored feedback based on triggers (1.9%) or pre-set goals (0.8%). Once again, the most common tools available were blood alcohol concentration calculators, applications that provided information, tracking calendars, and motivational applications. In summary, all three content analyses found that the alcohol applications available in stores were for either blood alcohol concentration calculation or entertainment. A similar focus on information or entertainment has also been observed in reviews of applications for cannabis use disorder; Ramo DE et al. (2015), using the search terminologies “cannabis” and “marijuana”, found 27.0% of identified applications to focus on the provision of information and 20.3% on entertainment [6]. Table 1 provides an overview of characteristics and key findings of each of the reviews.

Often, content analyses are limited to identification of functionalities in selected applications, but two of the three reviews of alcohol applications discussed above have included greater detail, with Crane D et al. (2015) identifying behavioral change techniques and Hoeppner BB et al. (2017) identifying applications that provided tailoring and customization of messages. This change in focus is seen in the recent content analyses of other types of smartphone applications. Morrissey EC et al. (2016) [7] reviewed existing medication adherence applications on the application stores and coded the identified applications based on the taxonomy of behavioural change. They found that behavioural change techniques, namely that of action planning, prompting, self-monitoring, and feedback on behaviour, to be commonly incorporated. Similarly, Ubhi HK et al. (2016) [8] also managed to identify common behavioural change techniques that have been incorporated in applications helping with smoking cessation. Some of the techniques include that of supporting identity change, rewarding abstinence, provision of advice on changing routines, and coping with cravings.

The change in the trends of content analyses since 2015 is pertinent. It is commonly known that interventions grounded in theory are complex, and thus, this would render them difficult to implement in research settings, especially on a smartphone device (Michie S et al., 2013) [9]. Very often, only certain elements of the complex theory are incorporated into an application. Behavioural change techniques have been defined as the “smallest, observable, replicable components with the potential to bring about change in behaviour” [5]. Thus, by identifying the behavioural change technique that has been included in an application, it helps to identify the complex underlying theory that the application is grounded in and helps to prove that the application is based on evidence. A taxonomy of 93 common behavioural change techniques has been identified previously by Michie S et al. (2013) [9]. These 93 behavioural techniques are clustered into 16 categories, which includes scheduled consequences, reward and threat, repetition and substitution, associations, covert learning, natural consequences, health consequences, feedback and monitoring, goals and planning, social support, comparison of behaviour, self-belief, comparison of outcomes, identity, shaping knowledge, and regulation.

## 3. Limitations

There is an increasing prevalence of other substance-related disorders, but the existing content analyses are limited to an analysis of alcohol and cannabis applications. The recent statistics released by the United Nations Office on Drugs and Crime have reported that at least a quarter of a billion individuals have experimented with substances in 2015, with the most commonly used drugs being that of cannabis, opioids, and amphetamines [10]. The annual prevalence of cannabinoid, opiate, and amphetamine usage are that of 3.8%, 0.7%, and 0.77%, respectively [10]. Given the prevalence of these disorders, it is thus imperative for us to perform content analyses of applications for other substance disorders to have a better understanding of commercial applications that are available.

Only two of the content analyses performed to date have attempted to identify applications that have their basis on a theoretical approach, based on the identification of behavioural change techniques, or motivational techniques. In contrast, most of the evaluated applications are conceptualised based on a theoretical approach. The “ACHESS” (Alcohol-Comprehensive Health Enhancement Support System) application has been conceptualised based on the self-determination theory, and it also helps equips individuals with cognitive behavioural skills for relapse prevention [10]. The LBMI-A (Location-Based Monitoring and Intervention System for Alcohol use disorders) is another example of an application that has its basis on theory, namely that of motivational enhancement, relapse prevention, and community reinforcement theories [11]. Within the LBMI-A application, the assessment function is a brief motivational intervention tool; the high-risk location functionality is based on principles of relapse prevention; the identification of supportive people is based on the Community Reinforcement and Family Training (CRAFT) approach, and the craving functionality based on cognitive behavioural theory [11]. The findings of a prior randomized trial involving the ACHESS application has reported that individuals who have had access to the application have had significantly fewer risky drinking days during a one-year period, whereas a pilot study involving the LBMI-A application helped individuals in reducing heavy drinking [11]. Given these findings, applications based on a theoretical approach could potentially be efficacious and useful clinically. Hence, there is a need to identify applications on the commercial stores that replicate conventional psychological interventions, or at least provide elements of conventional psychological interventions using the behavioural change techniques that are integrated into the application. Further evaluative research could be done on these applications to determine if they are efficacious before using them for patient care.

## 4. Bridging the Gaps

To address the limitation that existing content analyses have only focused on reviews of alcohol and cannabis applications, we propose for there to be updated content analyses for alcohol and cannabis, and new content analyses for other substances of abuse (such as opioids and stimulants). As discussed previously, the latest content analysis of alcohol applications was based on a cross-sectional sampling of the Android store in 2015 and the content analysis of cannabis applications was based on a cross-sectional sampling in 2014. Given how rapidly new applications are introduced onto these commercial stores, an updated content analysis would help to characterize the newer applications on these stores.

Any new content analyses of commercial applications should also aim to evaluate applications to determine if they have incorporated any behavioural change techniques. This would help to address the existing knowledge gap about the lack of identification of commercial applications that potentially have an evidence base. We recognised that most of the existing content analyses review tend to confine themselves to a defined number of applications on the application stores and have not reviewed what is available in its entirety. For example, in Crane D et al. (2015)’s review, only the initial 200 results were considered. It is postulated that individuals rarely go beyond the initial two screens on the application stores, and hence only a selected number of applications are routinely considered for further evaluation. By doing so, there is an inherent risk of missing out applications that might have incorporated evidence-based behavioural change techniques, as they are not within the predefined range of applications for review. The usage of new technologies, such as an application search engine could potentially mitigate against this risk, as it enables researchers to screen through applications quickly. Also, the consideration of more appropriate search terms could help in the identification of potentially more applications that has a basis on a behavioural change technique or a psychological theory. For the content analyses conducted to date, most of them are limited to the names of substances, such as “alcohol” or “cannabis” and permutations of these names. It was only in Hoeppner BB et al. (2017)’s review was there an additional term that of “sobriety” was used. We like to suggest that future reviews consider of keywords such as abstinence or recovery, and ones that relate to psychological therapies, such as self-determination or attention bias retraining, as commercial applications that have an underlying psychological basis might be categorised differently, under different keyword terms. We have evidence of how a better search strategy identifies previously unrecognised applications; when we searched both the Apple iTunes and Android Play store on 20 and 23 October 2017, using the following search terms: “attention bias” and “attention retraining”, we identified two applications that have been based on a psychological theoretical basis. To identify more applications based on attention bias modification, other keywords, such as that of “cognitive bias”, “approach bias”, “avoidance bias” could be used. Table 2 provides an overview of the two applications that we identified.

Our experiences suggest that if we were to expand the search terminologies to consider terminologies other than that of the substance, we would identify applications for addictive disorders, which are grounded in psychological theory and possibly more applications that have included elements of a behavioural change approach. Both the two applications identified in Table 2 are based on the principles of attention bias modification, which have been found to be useful for modification of attention biases in addictive disorders. Of significance, based on the statistics extracted from the Google Play store, both the identified applications have relatively low download rates, as well as a minimum number of ratings. These factors could have accounted for the fact as to why such applications, which are grounded in theory, have not been identified by prior searches. Of the two applications, “ChimpShop” [12] has been evaluated previously and was reported to be effective in reducing drinking amongst individuals with problem drinking by up to 60%. The existence of such applications on the commercial application stores highlights that there are available resources that individuals could easily tap onto, to help themselves in reducing the amount of alcohol they are consuming.

## 5. Conclusions

In conclusion, it is our view for there to be further reviews of substance-related applications and that these further reviews should aim to characterise existing applications and identify applications that have incorporated a behavioural change technique or have a basis on a psychological theory. Refinement of search strategy and considering all the available applications on the store for review might help in the identification of more applications that have incorporated an evidence base.

## Figures and Tables

**Table 1:** Characteristics and Key Findings of each Review.

Review Articles	Weaver ER et al. (2013) [4]	Crane D et al. (2015) [5]	Hoeppner BB et al. (2017) [2]	Ramo DE et al. (2015) [6]
**Main Substance Disorder**	Alcohol	Alcohol	Alcohol	Cannabis
**Keywords Used**	Alcohol	Alcohol	Drinking, Drink, Alcohol, Alcoholism, Sobriety	Cannabis, Marijuana
**Application Stores Searched**	Apple ITunes & Google Android Store	Apple ITunes & Google Android Store	Google Android Store	Apple ITunes & Google Android Store
**Number of applications included**	384 applications identified, but only the top 250 applications included for the review	Only initial 200 results considered, with 4 searches conducted, a total of 800 results considered. 662 applications included in final review	266 applications	342 applications from Apple App Store; 500 applications from Google Store
**Key Findings**	50% of applications were entertainment applications 11% provided information about health or methods to reduce drinking Analysis of blood alcohol concentration applications revealed these applications to be inaccurate	13.7% of applications helped in alcohol reduction 53.9% entertainment applications	60% of applications provided some mechanism for tailoring of feedback. Most common form of tailored feedback were triggers or pre-set goals. Most common tools were blood alcohol concentration calculators, applications that provided information, tracking calendars and motivational applications	27% of applications provided information 20.3% were entertainment applications

**Table 2:** Applications identified from commercial application stores that have basis on a psychological theory.

Name of App	App Description	App Functionalities	Underlying Psychological Theory	Total Number of Downloads (If Applicable)	Average Ratings
ChimpShop	ChimpShop contains psychological techniques to help make it easier for individuals to cut back on drinking.	Game, in which individuals would have to grab the “good stuff” and avoid the bad ones.	Attention Bias Modification	10–50 (on Google Play Store)	5.0 (based on 3 ratings)
Stay Sober, Stop Drinking	The simple process of consciously and repeatedly selecting non-alcoholic drinks such as water or orange juice over alcoholic beverages helps you to gradually develop a positive bias—a tendency to focus more on the positive information around you in everyday life.	Game, in which the intention is to avoid hitting the alcohol products	Attention Bias Modification	100–500 (on Google Play Store)	NA
